# Development and validation of risk factors prediction model for hypoxemia in the PACU patients after major abdominal surgery: a retrospective study

**DOI:** 10.3389/fmed.2026.1880924

**Published:** 2026-07-30

**Authors:** Min Zheng, Ya Fang, Zhengchun Kang

**Affiliations:** 1 Department of Anesthesiology, The First Affiliated Hospital of Naval Medical University, Shanghai, China; 2 Department of Colorectal Surgery, The First Affiliated Hospital of Naval Medical University, Shanghai, China

**Keywords:** hypoxemia, major abdominal surgery, nomogram, post-anesthesia care unit, retrospective study

## Abstract

**Objective:**

Hypoxemia is a common and potentially harmful complication in patients undergoing major abdominal surgery (MAS). We aimed to develop and validate a nomogram prediction model for hypoxemia in the post-anesthesia care unit (PACU) after MAS.

**Patients and methods:**

A total of 378 patients who underwent MAS and were admitted into the PACU were selected as the training group from June 1, 2024 to December 31, 2024. From April 1, 2025 to May 31, 2025, 189 patients who underwent MAS and were admitted to the PACU were enrolled as the validation group for temporal validation. Logistic regression analysis was employed to develop the nomogram model using age, body mass index (BMI), the ratio of arterial partial pressure of oxygen to fraction of inspired oxygen (PaO₂/FiO₂), and hemoglobin (HGB) as variables. The area under the receiver operating characteristic curve (AUC), calibration curves, and decision curve analysis (DCA) were used to evaluate the model accuracy. The primary outcome was defined as hypoxemia that occurred after tracheal extubation in the PACU.

**Results:**

In the training group (*n* = 378), hypoxemia occurred in 112 patients (29.6%). The final nomogram included four variables: age >60.5 years, BMI ≥ 24.0 kg/m^2^, PaO₂/FiO₂ < 311.5 mmHg, and HGB < 106.5 g/L (all *p* < 0.05). The model showed good calibration (Hosmer-Lemeshow test, *p* = 0.244) and discrimination (AUC = 0.88, 95% CI: 0.83–0.92). At the optimal cutoff of 0.242 (Youden index), sensitivity was 0.76 and specificity 0.85. Decision curve analysis demonstrated a positive net benefit across threshold probabilities of 0.03–1.00, confirming clinical utility. In the validation group (*n* = 189), the predictive accuracy was 78.3%, indicating that the model maintained excellent performance in the validation group.

**Conclusion:**

The nomogram model based on age, BMI, PaO₂/FiO₂, and HGB shows good predictive performance for hypoxemia. This model may help anesthesia staff identify patients at high risk of hypoxemia in the PACU.

## Introduction

Major Abdominal Surgery (MAS) refers to intra-abdominal procedures via open or minimally invasive approaches, involving resection of gastrointestinal, hepatobiliary, pancreatic, or renal organs (1). Laparoscopic surgeries with total incision length ≥5 centimeters (cm) are included, along with operation time expected to last >2 hours (h) or involved blood loss >500 milliliters (mL) (2, 3). A relevant study has confirmed that preoperative pulmonary function tests (including forced expiratory volume in one second, FEV₁) are associated with postoperative pulmonary complications (4). In addition, the patients undergoing MAS often experience restricted respiratory function due to factors like advanced age, preoperative comorbidities, significant surgical trauma, residual anesthetic effects, and postoperative pain, which can trigger hypoxemia (5, 6). The postoperative hypoxemia may lead to impaired wound healing, brain dysfunction, arrhythmias, myocardial ischemia, and even cardiopulmonary arrest (7, 8). Moreover, it prolongs post-anesthesia care unit (PACU) stays, increases costs, and consumes valuable medical resources (9–11). Emerging evidences suggest that signs of respiratory depression during early anesthesia recovery may increase the risk of severe adverse events (12).

Postoperative hypoxemia is one of the most common and potentially dangerous complications in the PACU. Some studies have investigated risk factors for PACU hypoxemia; however, most of these investigations have been restricted to specific surgical populations. Wang et al. (13) focused on patients with Stanford type A acute aortic dissection, a procedure requiring cardiopulmonary bypass with deep hypothermic circulatory arrest, where the mechanisms of postoperative hypoxemia are primarily driven by systemic inflammation and myocardial injury. Duan et al. (14) concentrated on elderly patients (≥65 years) with femoral neck fractures, in whom advanced age, reduced cardiopulmonary reserve, prolonged recumbency, and pulmonary infection are the main triggers of hypoxemia; moreover, that study simultaneously developed a dedicated nomogram prediction model. Li et al. (15) included pediatric patients undergoing general anesthesia; owing to developmental airway characteristics and a lower burden of baseline comorbidities, the risk factor profile for hypoxemia in children differs substantially from that in adults, and their study only performed risk factor analysis without establishing a predictive model. Taye et al. (9) and Andualem et al. (16) enrolled mixed adult surgical cohorts in high-altitude Ethiopian healthcare settings, where limited oxygen resources served as a unique confounding factor; both studies only screened for risk factors and did not develop predictive tools. To our knowledge, no studies have reported a predictive model of hypoxemia in PACU patients after MAS. In this study, we retrospectively analyzed patients undergoing MAS and aimed to construct a nomogram that clinicians can use to identify high-risk patients in the PACU.

## Patients and methods

### Study design and participants

**Figure 1:**
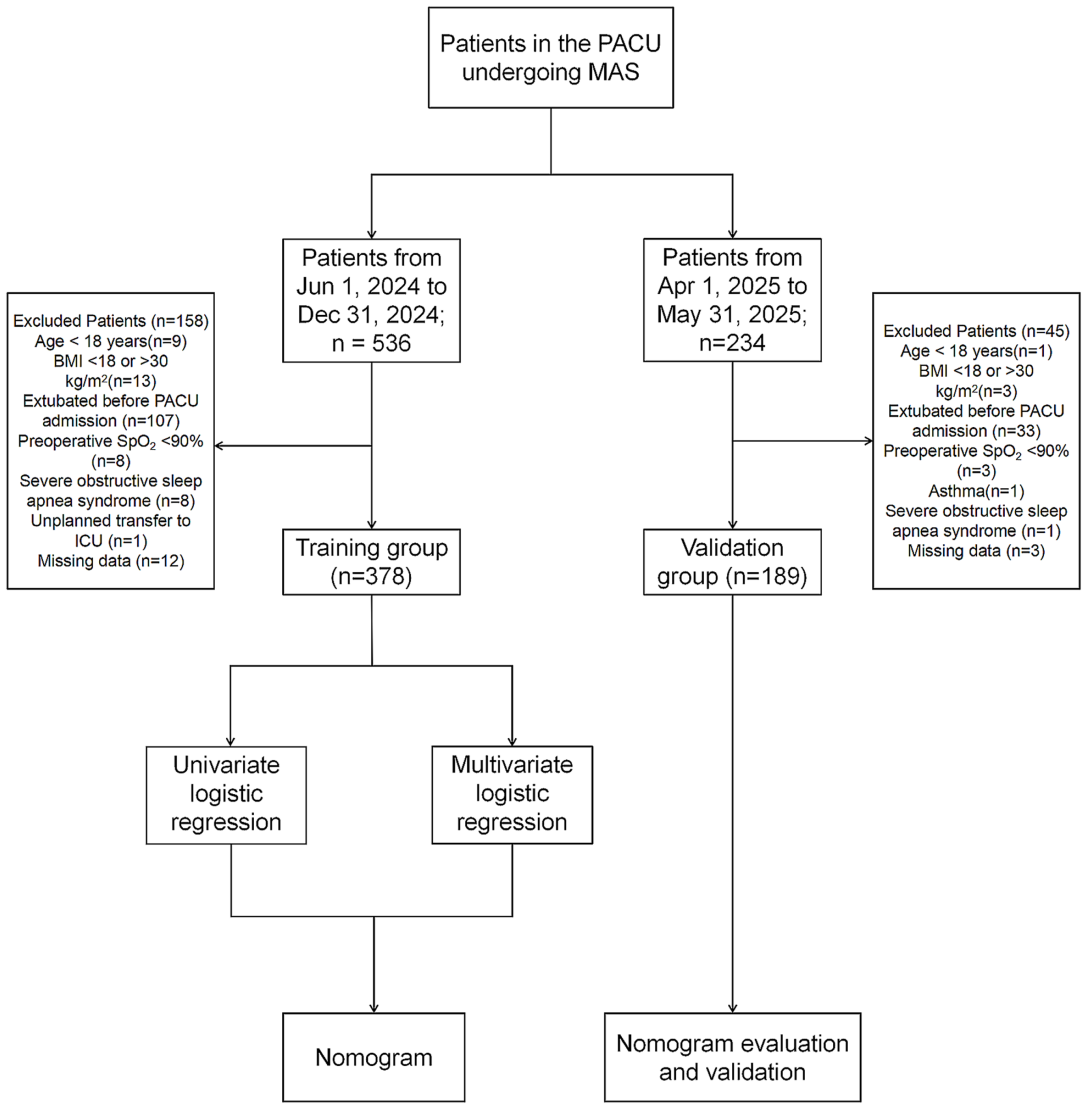
Flow diagram of the study.

All consecutive patients who met the inclusion criteria during the study period were enrolled. The training group included 378 patients treated between June 1, 2024, and December 31, 2024; the validation group included 189 patients treated between April 1, 2025, and May 31, 2025. The sample size was calculated using the events per variable (EPV) method, with EPV > 10 considered necessary to ensure stable estimates (17). The final multivariable model included 4 predictors. Based on the previous experiment, the incidence of hypoxemia in the PACU after MAS was approximately 33.0%. With EPV set at 10, the required sample size was calculated as: (4 × 10) /0.33 ≈ 121 cases. After adding 10% to account for missing data, approximately 134 cases were needed. The inclusion criteria for this study were as follows: (i) adults aged ≥18 years who underwent elective MAS under general anesthesia; (ii) BMI ranging from 18 to 30 kg/m^2^; (iii) American Society of Anesthesiologists (ASA) physical status II to III; and (iv) patients transported to the PACU with an endotracheal tube in place. Exclusion criteria were as follows: (i) unplanned transfer to the intensive care unit (ICU); (ii) preoperative severe respiratory disease (such as chronic obstructive pulmonary disease, asthma, respiratory failure, etc.) or pulse oximetry oxygen saturation (SpO_2_) less than 90% without oxygen inhalation; (iii) history of severe upper respiratory tract infection; (iv) severe obstructive sleep apnea syndrome (OSAS). The study was approved by the Ethics Committee of the First Affiliated Hospital of Naval Medical University (Approval No.: CHEC2025-317). The patient screening process is shown in Figure 1.

### Data collection and variables

Based on previous studies (9, 10) and reports from specialists, critical variables potentially influencing hypoxemia in PACU patients after MAS were selected, including demographic characteristics, intraoperative and postoperative data. The demographic characteristics included gender, age, BMI, smoking history, and underlying comorbidities such as hypertension, diabetes mellitus (DM), and coronary artery disease (CAD). Intraoperative and postoperative data included surgical approach, anesthesia duration, ASA physical status, total fluid volume (recorded in 500-mL increments, as each infusion bag contained 500 mL, rather than as a continuous variable), crystalloid/colloid fluid volume, blood loss volume, blood transfusion volume, sufentanil dosage, PaCO₂, PaO₂/FiO₂, HGB, potassium (K^+^), use of antagonistic drugs (neostigmine and atropine in a 1:1 ratio), and postoperative pain scores. The PaCO_2_, PaO_2_/FiO_2_, HGB, K^+^ were measured from an arterial blood gas sample obtained upon PACU arrival, before extubation. Postoperative pain was assessed after tracheal extubation in the PACU using the Numeric Rating Scale (NRS), ranging from 0 (no pain) to 10 (most severe pain) (18). All of the above data were obtained from the anesthesia record and arterial blood gas record. Variables with more than 20% missing data were excluded (preoperative FEV_1_, serum B-type natriuretic peptide, and postoperative additional opioid dose). In the PACU, hypoxemia was defined as SpO₂ < 90% post-extubation (19).

### Anesthesia management

Upon arrival in the operating room, routine monitoring, including electrocardiography (ECG), SpO₂, and non-invasive blood pressure (NIBP), was established, and pre-oxygenation was performed. Anesthesia was induced with sufentanil 0.2–0.5 μg/kg, propofol 2.0–2.5 mg/kg, and rocuronium 0.6 mg/kg or cis-atracurium 0.15 mg/kg, administered intravenously. Propofol was administered by slow intravenous titration over approximately 1 min; the injection was stopped when the patient lost consciousness (failed to respond to verbal commands), with blood pressure and heart rate continuously monitored throughout the induction process (20). In elderly patients, the same agents were administered by slow titration under continuous hemodynamic monitoring. Guidelines advise administration of weight-adjusted adequate neuromuscular blocking agents to facilitate tracheal intubation, minimize airway trauma and optimize intubating conditions (21). After loss of consciousness, tracheal intubation was performed, and mechanical ventilation was initiated with a tidal volume of 6–8 mL/kg of on predicted body weight, respiratory rate of 12–16 breaths/min, positive end-expiratory pressure (PEEP) of 5 cmH₂O, inspiratory-to-expiratory (I:E) ratio of 1:2, and end-tidal carbon dioxide (PETCO₂) target of 35–45 mmHg.

Anesthesia was maintained with continuous infusions of propofol (4–6 mg·kg^−1^·h^−1^) and remifentanil (0.1–0.5 μg·kg^−1^·min^−1^), with the bispectral index (BIS) maintained between 40 and 60. Intraoperative hemodynamic management followed a standardized protocol: if systolic blood pressure (SBP) fell below 90 mmHg or decreased by more than 25% from baseline, or if diastolic blood pressure (DBP) dropped below 50 mmHg, ephedrine hydrochloride 6 mg was administered intravenously; if SBP exceeded 180 mmHg or rose by more than 25% above baseline, or DBP exceeded 100 mmHg, urapidil 5 mg was given; if heart rate (HR) decreased to less than 45 beats/min or by more than 25% from baseline, atropine 0.5 mg was administered intravenously; if HR exceeded 120 beats/min or increased by more than 25% above baseline, esmolol 10 mg was given as a bolus.

At the end of surgery, all patients were transferred to the PACU with the endotracheal tube in place. The ventilator settings in the PACU were identical to those used during the intraoperative period (including tidal volume, respiratory rate, PEEP, I: E ratio, and PETCO₂ target), with synchronized intermittent mandatory ventilation (SIMV) mode applied to assist breathing. Vital signs were continuously monitored throughout the PACU stay. Extubation was performed strictly according to predefined criteria: (i) clear consciousness; (ii) recovery of muscle strength (strong hand grip, ability to maintain head lift for >3 s, and arm lift for >5 s); (iii) tidal volume 300–500 mL; (iv) regular breathing pattern; (v) normal PaCO₂; and (vi) regular thoracic cage movement. Following assessment by the anesthesiologist, the endotracheal tube was removed by the anesthesia nurse, and nasal cannula oxygen at 3 L/min was initiated.

Hypoxemia (SpO₂ < 90%) in the PACU was managed with a stepwise remedial protocol, including increasing oxygen flow rate, jaw-thrust manoeuvre, mask oxygen supplementation, and, if necessary, re-intubation and mechanical ventilation support, to ensure rapid improvement of oxygenation.

### Statistical analysis

Data were analyzed using SPSS 26.0 and R 4.0.3. Continuous variables were compared using Student’s t-test [mean ± standard deviation (SD)] or Mann–Whitney U test [median with interquartile range (IQR)]. Categorical variables were compared using Chi-square or Fisher’s exact test (percentages). Tolerance (TOL) and variance inflation factor (VIF) were employed to diagnose multicollinearity among independent variables with *p* < 0.05 in univariate analysis. Continuous variables were dichotomized using optimal cut-off values determined by the Youden index based on receiver operating characteristic (ROC) curves. A predictive nomogram model for hypoxemia was constructed based on the multivariate regression analysis. The Hosmer-Lemeshow (H-L) test was used to evaluate the goodness of fit of the nomogram model. The ROC and AUC were applied to evaluate the sensitivity and specificity of the hypoxemia prediction model. Discrimination and calibration were assessed using the bootstrap method (1,000 resamples) and calibration plots. DCA was applied to evaluate the clinical utility of the prediction model. Missing data: <5% imputed by mean (for normal distribution), median (for non-normal distribution), or mode (for categorical variables); 5–20% by multiple imputation; >20% excluded. To balance baseline characteristics between cohorts, 1∶1 propensity score matching (PSM) was performed with a caliper of 0.02, and balance was assessed using standardized mean differences (SMD < 0.2 indicating good balance). All tests were two-sided with *p* < 0.05 considered significant.

## Results

### Clinical characteristics of patients

The incidence of postoperative hypoxemia in the training group was 29.6% (112 out of 378 patients). In the training group, the patients’ mean age was 56.8 ± 19.0 years, 214 (56.6%) were male, and mean BMI was 22.6 ± 3.0 kg/m^2^. In the validation group, the patients’ mean age was 58.6 ± 16.1 years, 112 (59.3%) were male, and the mean BMI was 22.8 ± 2.8 kg/m^2^. The incidence of postoperative hypoxemia in the validation group was 28.0% (53 out of 189 patients). Before PSM, significant differences were observed between the two groups in PaCO_2,_ surgical approach, smoking history, and K^+^ (all *p* < 0.05). After 1∶1 PSM, 165 pairs were successfully matched. After matching, the SMDs for these four variables were 0.021, 0.075, 0.037, and 0.122, respectively, all below the acceptable threshold of 0.2 (Supplementary Table 1). Univariate analysis showed that age, BMI, smoking history, PaO₂/FiO₂, HGB, and postoperative pain score were statistically significant in both the training and the validation groups (*p* < 0.05; Table 1).

**Table 1:** Univariate analysis of hypoxemia group and nonhypoxemia group in the PACU patients after MAS in the training and validation groups.

Variables	Training group (*n* = 378)		Statistic	*p*	Validation group (*n* = 189)		Statistic	*p*
	Nonhypoxemia (*n* = 266)	Hypoxemia (*n* = 112)			Nonhypoxemia (*n* = 136)	Hypoxemia (*n* = 53)		
Demographic data								
Age (years), Mean ± SD	54.4 ± 20.0	62.6 ± 15.0	t = −3.08	0.002	57.0 ± 17.0	62.6 ± 12.9	t = −2.12	0.035
BMI(kg/m^2^), Mean ± SD	21.8 ± 2.4	24.5 ± 3.4	t = −8.88	<0.001	22.1 ± 2.5	24.5 ± 3.0	t = −5.11	<0.001
Male, *n* (%)	155 (58.3)	59 (52.7)	χ^2^ = 1.08	0.298	80 (58.8)	32 (60.4)	χ^2^ = 0.12	0.906
Hypertension, *n* (%)	68 (25.6)	22 (19.6)	χ^2^ = 1.42	0.233	33 (24.3)	19 (35.9)	χ^2^ = 2.47	0.116
DM, *n* (%)	43 (16.2)	24 (21.4)	χ^2^ = 1.70	0.193	28 (20.6)	13 (24.5)	χ^2^ = 0.32	0.572
CAD, *n* (%)	6 (2.3)	7 (6.3)	χ^2^ = 2.74	0.098	5 (3.7)	1 (1.9)	χ^2^ = 0.03	0.866
Smoking, *n* (%)	181 (68.1)	58 (51.8)	χ^2^ = 8.70	0.003	81 (59.6)	22 (41.5)	χ^2^ = 5.01	0.025
Intraoperative data								
ASA, *n* (%)			χ^2^ = 2.53	0.116			χ^2^ = 1.89	0.169
Grade II	208 (78.2)	79 (70.5)			110 (80.9)	38 (71.7)		
Grade III	58 (21.8)	33 (29.5)			26 (19.1)	15 (28.3)		
Surgical approach, *n* (%)			χ^2^ = 1.04	0.309			χ^2^ = 0.00	0.949
Open surgery	134 (50.4)	50 (44.6)			52 (38.2)	20 (37.7)		
Laparoscopic surgery	132 (49.6)	62 (55.4)			84 (61.8)	33(62.3)		
Anesthesia duration (min), median (IQR)	195.0 (165.0, 220.0)	195.0 (165.0, 215.8)	Z = −0.25	0.806	185.0 (155.0, 215.0)	190.0 (175.0, 220.0)	Z = −1.38	0.167
Fluid volume (mL), median (IQR)	2000.0 (2000.0, 2500.0)	2000.0 (1500.0, 2000.0)	Z = −1.73	0.084	2000.0 (1500.0, 2500.0)	2000.0 (2000.0, 2500.0)	Z = −1.13	0.258
Crystalloid volume (mL), median (IQR)	1100.0 (1000.0, 1500.0)	1100.0 (1000.0, 1500.0)	Z = −1.22	0.221	1000.0 (1000.0, 1500.0)	1000.0 (1000.0, 1500.0)	Z = −0.31	0.758
Colloid volume (mL), median (IQR)	1000.0 (500.0, 1000.0)	1000.0 (500.0, 1000.0)	Z = −0.29	0.775	1000.0 (500.0, 1000.0)	1000.0 (500.0, 1000.0)	Z = −1.56	0.119
Blood loss (mL), median (IQR)	100.0 (50.0, 200.0)	100.0 (50.0, 200.0)	Z = −1.64	0.100	100.0 (50.0, 200.0)	100.0 (50.0, 200.0)	Z = −0.37	0.708
Sufentanil dosage (μg), median (IQR)	15.0 (0.0, 25.0)	15.0 (0.0, 25.6)	Z = −0.40	0.691	15.0 (10.0, 25.6)	15.0 (10.0, 25.0)	Z = −0.62	0.538
Blood transfusion volume (mL), median (IQR)	0.0 (0.0, 0.0)	0.0 (0.0, 0.0)	Z = −0.38	0.703	0.0 (0.0, 0.0)	0.0 (0.0, 0.0)	Z = −1.72	0.086
PaCO_2_ (mmHg), median (IQR)	40.0 (35.0, 44.0)	39.0 (34.8, 44.0)	Z = −0.70	0.482	42.0 (38.0, 45.3)	41.0 (38.0, 46.0)	Z = −0.10	0.923
PaO_2_/FiO_2_ (mmHg), median (IQR)	512.0 (362.8, 561.0)	210.0 (161.8, 290.3)	Z = −10.28	<0.001	470.5 (291.8, 525.0)	281.0 (161.0, 508.0)	Z = −3.92	<0.001
HGB (g/L), median (IQR)	128.0 (115.0, 139.0)	103.0 (89.8, 123.3)	Z = −8.89	<0.001	126.5 (117.0, 136.3)	113.0 (100.0, 127.0)	Z = −4.27	<0.001
K^+^ (mmol/L), median (IQR)	4.1 (3.7, 4.4)	4.0 (3.8, 4.2)	Z = −0.77	0.442	3.8 (3.6, 4.2)	3.9 (3.6, 4.3)	Z = −1.64	0.101
Postoperative data								
Use of antagonistic drugs, *n* (%)	57 (21.4)	34 (30.4)	χ^2^ = 3.44	0.064	31 (22.8)	15 (28.3)	χ^2^ = 0.63	0.428
Postoperative pain score, median (IQR)	3.0 (2.0, 3.0)	4.0 (2.0, 5.0)	Z = −8.25	<0.001	3.0 (2.0, 3.0)	3.0 (2.0, 4.0)	Z = −1.98	0.048

PACU, post-anesthesia care unit; MAS, major abdominal surgery; SD, standard deviation; BMI, body mass index; DM, diabetes mellitus; CAD, coronary artery disease; ASA, American Society of Anesthesiologists; IQR, interquartile range; PaCO₂, partial pressure of carbon dioxide; PaO₂, partial pressure of oxygen; FiO₂, fraction of inspired oxygen; HGB, hemoglobin; K^+^, potassium.

### Model development

The univariate analysis showed that six independent variables were statistically significant. Collinearity diagnostics indicated that the TOL values were all >0.1, and the VIF values were 1.124, 1.015, 1.189, 1.145, 1.346, and 1.238, respectively, which could therefore be included in the multivariate analysis. To simplify the model and enhance its clinical utility, we converted age (>60.5 years), BMI (≥24.0 kg/m^2^), PaO₂/FiO₂ (<311.5 mmHg), and HGB (<106.5 g/L) into categorical variables based on the optimal cut-off values determined by the Youden index. The logistic regression analysis based on these critical variables showed that age >60.5 years [odds ratio (OR): 3.01, 95% CI: 1.48–6.13, *p* = 0.002], BMI ≥ 24.0 kg/m^2^ (OR: 5.28, 95% CI: 2.60–10.71, *p* < 0.001), PaO₂/FiO₂ < 311.5 mmHg (OR: 25.00, 95% CI: 11.11–49.98, *p* < 0.001), and HGB < 106.5 g/L (OR: 12.50, 95% CI: 5.56–25.00, *p* < 0.001) were independent predictors of the development of postoperative hypoxemia (Table 2). According to the results of the multivariable analysis, the predictive formula of hypoxemia was established as follows: Logit (*P*) = 1.14 + 1.10 × (Age >60.5 years) + 1.66 × (BMI ≥ 24.0 kg/m^2^) + 3.14 × (PaO₂/FiO₂ < 311.5 mmHg) + 2.52 × (HGB < 106.5 g/L).

**Table 2:** Multivariable logistic regression analysis of hypoxemia in PACU patients after MAS (training group, *n* = 378).

Variables	β	S. E	Z	*p*	OR (95%CI)
Intercept	1.14	0.45	2.54	0.011	3.13 (1.30–7.55)
Age >60.5 years	1.10	0.36	3.03	0.002	3.01 (1.48–6.13)
BMI ≥ 24.0 kg/m^2^	1.66	0.36	4.61	<0.001	5.28 (2.60–10.71)
Smoking	0.10	0.24	0.41	0.683	1.10 (0.69–1.75)
PaO_2_/FiO_2_ < 311.5 mmHg	3.14	0.39	8.15	<0.001	25.00 (11.11–49.98)
HGB < 106.5 g/L	2.52	0.40	6.31	<0.001	12.50 (5.56–25.00)
Postoperative pain score	0.12	0.07	1.67	0.096	1.13 (0.98–1.30)

PACU, post-anesthesia care unit; MAS, major abdominal surgery; OR, odds ratio; CI, confidence interval; BMI, body mass index; PaO₂, partial pressure of oxygen in arterial blood; FiO₂, fraction of inspired oxygen; HGB, hemoglobin.

### Nomogram model display

**Figure 2:**
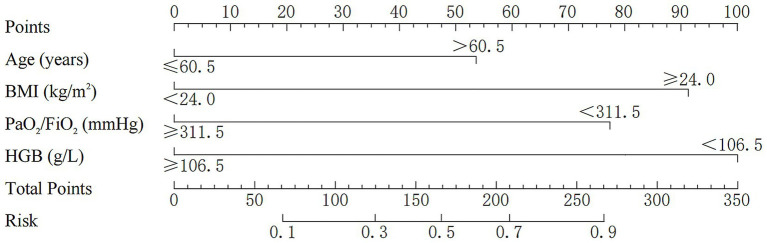
Nomogram to predict the risk of hypoxemia in PACU patients after MAS. Each vertical line is used to assign points for each variable. The total number of points is then calculated, and a vertical line is drawn downward from the total point row to obtain the probability of postoperative hypoxemia. PACU, post-anesthesia care unit; MAS, major abdominal surgery; BMI, body mass index; PaO₂, partial pressure of oxygen in arterial blood; FiO₂, fraction of inspired oxygen; HGB, hemoglobin.

For risk factor prediction of postoperative hypoxemia, we constructed a nomogram prediction model based on the multivariable logistic regression results (Figure 2). To use the nomogram, a vertical line was drawn up to the top point row to assign points for each variable. The total number of points was then calculated, and a vertical line was drawn downward from the total point row to obtain the probability of postoperative hypoxemia.

### Nomogram evaluation and validation

**Figure 3:**
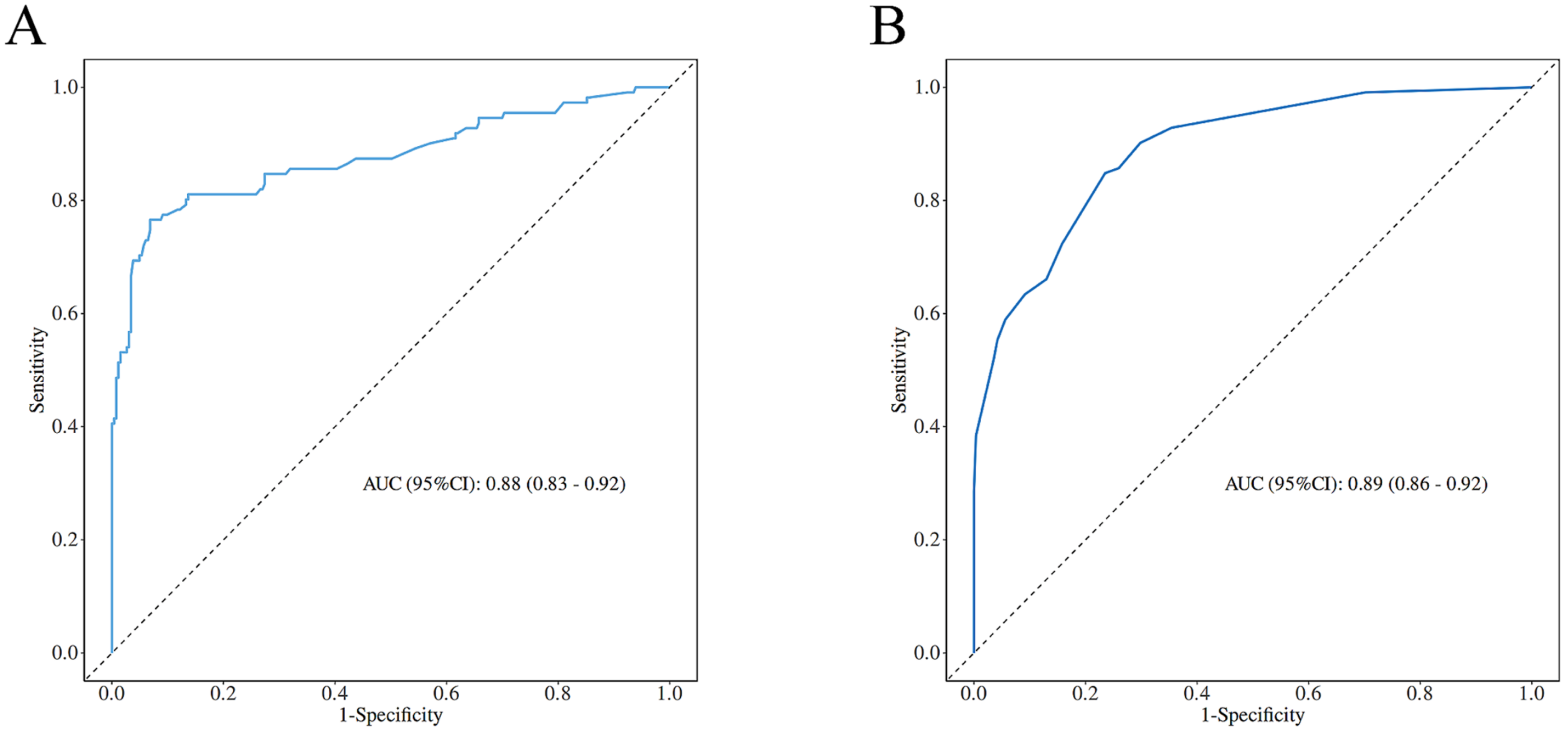
AUC of the model for predicting the risk of hypoxemia in PACU patients after MAS. **(A)** Training group. **(B)** Validation group. The blue curve represents the ROC curve of the nomogram. The optimal cut-off point (Youden index) is indicated by the dot on the curve. ROC, receiver operating characteristic curve; AUC, area under the curve.

**Figure 4:**
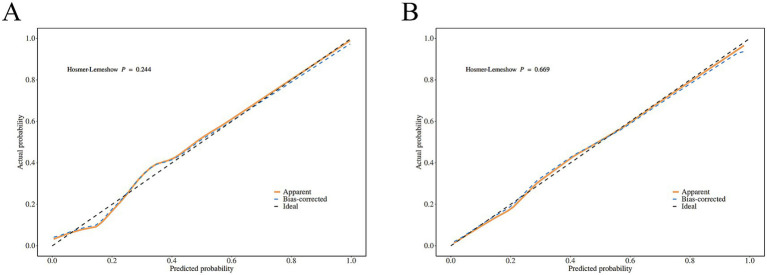
Calibration curve of the nomogram for the training group **(A)** and the validation group **(B)**. The bias-corrected curve was generated using bootstrap internal validation with 1,000 resamples to reduce overfitting optimism. The black dotted line represents the ideal prediction, while the orange line represents the actual calibration curve of the nomogram. The blue line represents the internally corrected calibration curve of the nomogram.

**Figure 5:**
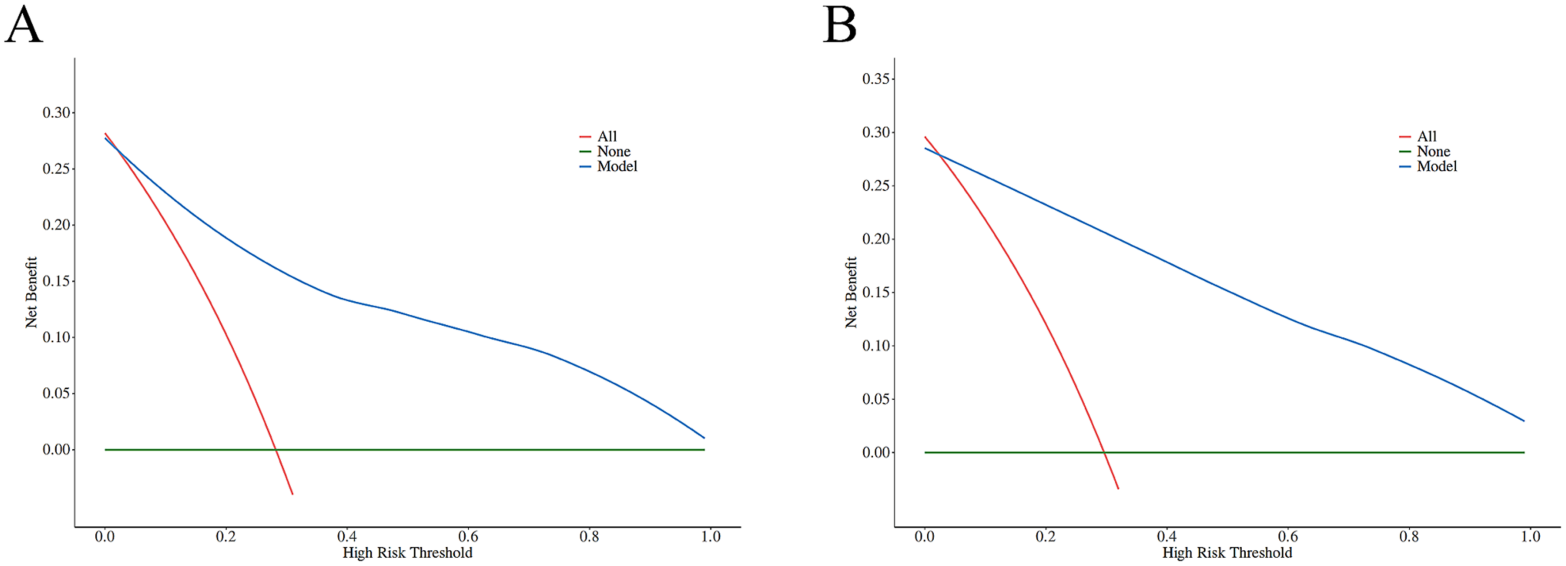
Decision curve analysis of the nomogram for the risk of hypoxemia in PACU patients after MAS. **(A)** Training group. **(B)** Validation group. The green line indicates extreme cases in which the model predicts that all patients have a low hypoxemia probability, with a clinical net benefit of 0. The red curve indicates extreme cases in which the model predicts that all PACU patients after MAS have a moderate or high hypoxemia probability, with a negative slope of clinical net benefit. The blue line indicates that the model yields clinical net benefit. PACU, post-anesthesia care unit; MAS, major abdominal surgery.

To evaluate the predictive performance of the nomogram, we calculated the AUC using the predicted probability from the model. The AUC was 0.88 (95% CI: 0.83–0.92; Figure 3A). Using the Youden index, the optimal probability cutoff was determined as 0.242, at which the sensitivity was 0.76 and the specificity was 0.85. The prediction model was internally validated via bootstrap resampling (1,000 resamples), and a calibration plot was constructed to compare predicted and observed hypoxemia outcomes (Figure 4A). The H-L test yielded a *p* value of 0.244. DCA showed that, in the training group, the model provided a higher net benefit than the “all intervention” or “no intervention” strategies across a threshold probability range of 0.03–1.00 (Figure 5A).

In the validation group, the AUC was 0.89 (95% CI: 0.86–0.92; Figure 3B), and the H-L test showed *p* = 0.669 (Figure 4B). Among 189 patients, the model predicted 64 positive cases, while the actual number of hypoxemia events was 53. Correctly predicted positive cases were 38, yielding a sensitivity of 71.7%. Predicted negative cases were 125, while the actual number of non-hypoxemia cases was 136; correctly predicted negative cases were 110, giving a specificity of 80.9%. The overall predictive accuracy was therefore (38 + 110)/189 ≈ 78.3%. DCA for the validation group showed a higher net benefit than the “all intervention” or “no intervention” strategies across a threshold probability range of 0.04–1.00 (Figure 5B).

## Discussion

The postoperative hypoxemia incidence in our MAS cohort (29.6%) falls within the broad range of 4.35–45.8% reported previously (9, 13, 15, 16, 22). This wide variation reflects the combined effects of age, BMI, comorbidity burden, surgical type, hypoxemia definition, and geographic setting. Duan et al. (14) reported an incidence of 30.23% in elderly hip fracture patients (mean age 79 years, 36.5% ASA III-IV), close to our 29.6% (mean age 56.8 years, 24.1% ASA III). Although our cohort was younger and had fewer high‑risk ASA patients, the incidence remained similar, possibly because their stricter definition (PaO₂/FiO₂ ≤ 300 mmHg) counterbalanced their higher baseline risk, whereas we used SpO₂ < 90%.

Wang et al. (13) studied aortic dissection patients and identified serum angiotensin II, BMI, and cardiac troponin I as independent risk factors, reporting an incidence of 28.4%—slightly lower than ours—likely due to their more stringent cutoff of PaO₂/FiO₂ ≤ 200 mmHg. Andualem et al. (16) reported a lower incidence of 24.5% in a younger cohort (mean age 37.6 years, 2.3% ASA III), and identified age >55 years as an independent risk factor, which aligns with our finding that age >60.5 years was an independent predictor. Taye et al. (9) reported the highest rate (45.8%) in a high-altitude Ethiopian setting (2,706 m), where 85.4% of patients received no supplemental oxygen during transfer—and the absence of oxygen therapy was identified as an independent predictor. In contrast, our low-altitude setting (≈4 m) with standardized oxygen supplementation during transport may explain the substantially lower incidence in our cohort.

In our study, we developed a nomogram to predict the risk of hypoxemia in the PACU following MAS. Based on the multivariate logistic regression analysis, age >60.5 years, BMI ≥ 24.0 kg/m^2^, PaO₂/FiO₂ < 311.5 mmHg, and HGB < 106.5 g/L were identified as risk factors for postoperative hypoxemia. Firstly, older age has been reported to be associated with the development of hypoxemia in various surgeries (14, 16, 22, 23), which was consistent with the results of this study (OR = 3.01, 95% CI: 1.48–6.13, *p* = 0.002). Chern et al. (24) reported that advanced age is a risk factor for postoperative pulmonary complications due to a decline in physical function and increased comorbidities. These factors may weaken respiratory muscle strength and reduce lung tissue elasticity, leading to decreased vital capacity and functional residual capacity, and thereby inducing hypoxemia. For elderly patients undergoing MAS, performing lung recruitment maneuvers (LRM) before extubation and applying 5 cmH₂O of PEEP can reduce postoperative atelectasis and hypoxemia, and decrease the incidence of oxygen desaturation and oxygen requirements (25). In addition, patients with BMI ≥ 24.0 kg/m^2^ had 5.28 times higher odds of hypoxemia than those with BMI < 24.0 kg/m^2^ (OR = 5.28, 95% CI: 2.60–10.71, *p* < 0.001). Multiple previous studies have reported a positive correlation between increasing BMI and hypoxemia risk, which is consistent with the findings of this study (9, 13, 22, 23, 26). Patients with a BMI ≥ 24.0 kg/m^2^ in this study met the diagnostic criteria for overweight based on the Chinese classification. The possible mechanisms of hypoxemia in overweight/obese patients are as follows. In these patients, pulmonary fibrosis caused by mechanical injury, inflammatory mediators (such as TNF-*α*, TGF-*β*, and MCP-1) produced by excessive adipose tissue and infiltrating immune cells, as well as factors like vitamin D deficiency, insulin resistance, and oxidized low-density lipoprotein, lead to decreased lung compliance and thus impair respiratory function (27). Studies have confirmed that obesity activates pulmonary macrophages and inflammatory pathways, exacerbating pulmonary inflammatory responses and tissue damage (28). Additionally, overweight/obese patients with comorbid metabolic syndrome and cardiovascular diseases show increased intraoperative oxygen demand and insufficient oxygen reserve. Aggravated respiratory workload during postoperative recovery further predisposes them to hypoxemia (29). Studies have reported that obesity is associated with an increased risk of postoperative pulmonary complications, and that high-flow nasal cannula (HFNC) applied in the PACU for overweight/obese patients can effectively reduce pulmonary complications including hypoxemia (30).

This study found that a PaO₂/FiO₂ ratio <311.5 mmHg was a risk factor for postoperative hypoxemia (OR: 25.00, 95% CI: 11.11–49.98, *p* < 0.001), aligning with Duan et al.’s conclusion that a preoperative PaO₂/FiO₂ < 300 mmHg is an independent risk factor (14). A PaO₂/FiO₂ below 300 mmHg indicates acute lung injury (ALI) or acute respiratory distress syndrome (ARDS), leading to alveolar collapse and decreased lung compliance. Consequently, the ventilation-perfusion ratio becomes imbalanced, triggering hypoxemia (31). A PaO₂/FiO₂ < 311.5 mmHg indicates potential abnormalities in pulmonary oxygenation, reflecting reduced gas exchange efficiency and poor tolerance to surgical trauma and anesthesia. To reduce hypoxemia risk, we suggest: preoperatively identify high-risk patients (PaO₂/FiO₂ < 311.5) and optimize respiratory function; intraoperatively use lung-protective ventilation; and in the PACU, closely monitor oxygenation and promptly administer high-flow nasal oxygen or noninvasive ventilation when clinically indicated (32). Studies have reported that patients undergoing MAS can mitigate alveolar overdistension and relieve pressure/volume injuries through low tidal volume ventilation, and “open the lungs” via PEEP and/or LRM to reduce atelectasis-related injuries, thereby decreasing postoperative pulmonary complications (33). In addition, research has reported that for obese patients with a low oxygenation index, postoperative non-invasive respiratory support with continuous positive airway pressure (CPAP) is an optimal strategy to improve oxygenation (34).

Finally, HGB levels are closely linked to oxygen-carrying capacity. When HGB was <106.5 g/L, postoperative hypoxemia was more likely to occur (OR: 12.50, 95% CI: 5.56–25.00, *p* < 0.001), which is similar to previous findings showing significant differences in mean HGB levels between hypoxemic and non-hypoxemic COVID-19 patients (35). As previous research has pointed out, the body relies on sufficient circulating HGB to continuously transport oxygen. Insufficient HGB impairs oxygen delivery, directly contributing to the development of hypoxemia (36). MAS frequently leads to blood loss, fluid shifts, and inflammatory responses, inducing perioperative anemia, which causes compensatory tachypnea and increases the risk of respiratory muscle fatigue. In addition, postoperative tissue hypoxia in anemic patients can trigger reflex pulmonary vasoconstriction, aggravate ventilation/perfusion mismatch, and further exacerbate hypoxemia. Therefore, preoperative anemia correction, rational intraoperative blood transfusion, and optimized postoperative oxygen therapy protocols are crucial for improving patients’ oxygenation status.

Notably, statistically significant differences were observed between the training and validation groups in several baseline characteristics, including PaCO_2_, surgical approach, smoking history, and serum potassium levels (*p* < 0.05). This discrepancy primarily arises from the fact that the two cohorts were derived from different time periods, reflecting the natural heterogeneity of patient populations over time in clinical practice. After PSM, all covariates had SMDs < 0.2, indicating good comparability. The model also maintained good predictive performance in the independent validation group (AUC = 0.89, 95% CI: 0.86–0.92; H-L test, *p* = 0.669; overall accuracy 78.3%). In the training group, the model achieved an AUC of 0.88 (95% CI: 0.83–0.92), with good calibration indicated by the H-L test (*p* = 0.244), and the calibration curves showed close agreement between predicted and observed outcomes. Using the optimal cutoff of 0.242 determined by the Youden index, the sensitivity and specificity in the training group were 0.76 and 0.85, respectively. DCA demonstrated that the nomogram provided a higher net benefit than either the “all intervention” or “no intervention” strategies across a wide range of threshold probabilities, confirming its clinical utility. Collectively, these results indicate that the nomogram possesses good discrimination, calibration, and clinical net benefit, supporting its potential as a practical tool for preoperative risk assessment. Anesthesia providers can use this nomogram to quantify the probability of post-extubation hypoxemia in PACU patients undergoing MAS, thereby identifying high-risk patients who may benefit from closer monitoring.

### Limitations

Several limitations should be acknowledged. First, this was a single-center retrospective study. Despite propensity score matching to balance baseline discrepancies between temporally separated training and validation groups, unmeasured confounders may remain. Second, hypoxemia was identified by pulse oximetry rather than routine post-extubation arterial blood gas testing, which may not accurately reflect actual arterial oxygenation. Third, we excluded patients with preoperative chronic respiratory disorders and severe OSAS to reduce confounding, so our model cannot be directly generalized to these high-risk populations. Fourth, we failed to analyze the interaction between postoperative NRS pain scores and analgesic administration, owing to inconsistent timing of intraoperative sufentanil dosage recording and postoperative pain score collection, as well as a missing rate exceeding 20% for records of additional analgesics administered in the PACU; besides, other key indicators including preoperative FEV_1_ and serum B-type natriuretic peptide were also discarded for excessive missing values. Though multiple imputation was used for variables with mild missingness, critical predictive information might be lost. These limitations underline the necessity of large prospective multicenter trials to verify our results.

## Conclusion

We developed and validated a new nomogram to predict the risk of hypoxemia in PACU patients undergoing MAS, based on four independent risk variables: age >60.5 years, BMI ≥ 24.0 kg/m^2^, PaO₂/FiO₂ < 311.5 mmHg, and HGB < 106.5 g/L. The nomogram may assist medical staff in identifying patients at high risk for hypoxemia in the PACU.

## Data Availability

The original contributions presented in the study are included in the article/Supplementary material, further inquiries can be directed to the corresponding author.

## Glossary

ALI: acute lung injury
ARDS: acute respiratory distress syndrome
ASA: American Society of Anesthesiologists
AUC: area under the curve
BIS: bispectral index
BMI: body mass index
CAD: coronary artery disease
CI: confidence interval
CPAP: continuous positive airway pressure
DCA: decision curve analysis
DBP: diastolic blood pressure
DM: diabetes mellitus
EPV: events per variable
FEV₁: forced expiratory volume in one second
FiO₂: fraction of inspired oxygen
HGB: hemoglobin
H-L test: Hosmer–Lemeshow test
HR: heart rate
I: E: inspiratory-to-expiratory
IQR: interquartile range
LRM: lung recruitment maneuver
MAS: major abdominal surgery
NRS: Numeric Rating Scale
OR: odds ratio
OSAS: obstructive sleep apnea syndrome
PACU: post-anesthesia care unit
PaCO₂: partial pressure of carbon dioxide in arterial blood
PaO₂: partial pressure of oxygen in arterial blood
PEEP: positive end-expiratory pressure
PETCO₂: end-tidal carbon dioxide
PSM: propensity score matching
ROC: receiver operating characteristic
SBP: systolic blood pressure
SD: standard deviation
SIMV: synchronized intermittent mandatory ventilation
SMD: standardized mean difference
SpO₂: peripheral oxygen saturation
TOL: tolerance
VIF: variance inflation factor.
