# Supplementary material for: Development and validation of risk factors prediction model for hypoxemia in the PACU patients after major abdominal surgery: a retrospective study
Source: Front Med (Lausanne). 2026 Jul 30;13:1880924. doi: 10.3389/fmed.2026.1880924 (PMC13469640; doi:10.3389/fmed.2026.1880924)
Supplement: Supplementary file 1 [file Table_1.doc]

**Supplementary Table 1.** Comparison of baseline characteristics between the two groups before and after propensity score matching (PSM)

|  | Before PSM | | | | | | After PSM | | | | | |
| --- | --- | --- | --- | --- | --- | --- | --- | --- | --- | --- | --- | --- |
| Variables | Total  (n = 567) | training group  (n = 378) | validation group  (n = 189) | Statistic | *P* | SMD | Total  (n = 330) | training group  (n = 165) | validation group  (n = 165) | Statistic | *P* | SMD |
| **Demographic data** | | | | | |  |  |  |  |  |  |  |
| Age, Mean ± SD | 57.4 ± 18.1 | 56.8 ± 19.0 | 58.6 ± 16.1 | t=-0.21 | 0.836 | 0.019 | 57.0 ± 17.5 | 59.1 ± 15.8 | 58.4 ± 16.4 | t=0.414 | 0.679 | -0.045 |
| BMI, Mean ± SD | 22.67 ± 2.94 | 22.61 ± 3.01 | 22.79 ± 2.81 | t=-0.68 | 0.497 | 0.064 | 22.7 ± 2.9 | 22.7 ± 3.1 | 22.7 ± 2.8 | t=-0.088 | 0.930 | 0.010 |
| Male, n (%) | 326 (57.50) | 214 (56.61) | 112 (59.26) | χ²=0.32 | 0.571 | 0.051 | 191 (57.88) | 95 (57.58) | 96 (58.18) | χ²=0.012 | 0.911 | 0.012 |
| Hypertension, n(%) | 142 (25.04) | 90 (23.81) | 52 (27.51) | χ²=1.16 | 0.282 | 0.093 | 88 (26.67) | 45 (27.27) | 43 (26.06) | χ²=0.062 | 0.803 | -0.028 |
| DM, n(%) | 108 (19.05) | 67 (17.72) | 41 (21.69) | χ²=1.29 | 0.256 | 0.097 | 60 (18.18) | 27 (16.36) | 33 (20.00) | χ²=0.733 | 0.392 | 0.091 |
| CAD, n(%) | 19 (3.35) | 13 (3.44) | 6 (3.17) | χ²=0.03 | 0.865 | 0.016 | 9 (2.73) | 3 (1.82) | 6 (3.64) | χ²=0.457 | 0.499 | 0.097 |
| Smoking, n(%) | 342 (60.32) | 239 (63.23) | 103 (54.50) | χ²=4.33 | 0.037 | -0.182 | 193 (58.48) | 98 (59.39) | 95 (57.58) | χ²=0.112 | 0.738 | -0.037 |
| **Intraoperative data** | | | | | |  |  |  |  |  |  |  |
| ASA, n(%) |  |  |  | χ²=0.42 | 0.517 |  |  |  |  | χ²=0.158 | 0.691 |  |
| Grade II | 435 (76.72) | 287 (75.93) | 148 (78.31) |  |  | 0.058 | 257 (77.88) | 130 (78.79) | 127 (76.97) |  |  | -0.043 |
| Grade III | 132 (23.28) | 91 (24.07) | 41 (21.69) |  |  | -0.058 | 73 (22.12) | 35 (21.21) | 38 (23.03) |  |  | 0.043 |
| Surgical approach, n(%) |  |  |  | χ²=5.402 | 0.020 |  |  |  |  | χ²=0.457 | 0.499 |  |
| Open surgery | 256 (45.15) | 184 (48.68) | 72 (38.10) |  |  | -0.212 | 130 (39.39) | 68 (41.21) | 62 (37.58) |  |  | -0.075 |
| Laparoscopic surgery | 311 (54.85) | 194(51.32) | 117 (61.90) |  |  | -0.212 | 200 (60.61) | 97 (58.79) | 103 (62.42) |  |  | 0.075 |
| Anesthesia duration (min), median (IQR) | 195.00 (161.00, 219.00) | 195.00 (165.00, 220.00) | 185.00 (158.00, 215.00) | Z=-0.80 | 0.424 | -0.056 | 190.00 (160.00, 215.00) | 195.00 (165.00, 215.00) | 189.00 (160.00, 215.00) | Z=-0.234 | 0.815 | 0.054 |
| Fluid volume (ml), median (IQR) | 2000.00 (2000.00, 2500.00) | 2000.00 (2000.00, 2500.00) | 2000.00 (1600.00, 2500.00) | Z=-0.04 | 0.970 | 0.001 | 2000.00 (2000.00, 2500.00) | 2000.00 (1600.00, 2500.00) | 2000.00 (2000.00, 2500.00) | Z=-0.016 | 0.987 | 0.018 |
| Crystalloid volume (ml), median (IQR) | 1000.00 (1000.00, 1500.00) | 1000.00 (1000.00, 1500.00) | 1000.00 (1000.00, 1500.00) | Z=-1.24 | 0.214 | 0.195 | 1000.00 (1000.00, 1500.00) | 1000.00 (1000.00, 1500.00) | 1000.00 (1000.00, 1500.00) | Z=-0.390 | 0.697 | -0.078 |
| Colloid volume (ml), median (IQR) | 1000.00 (500.00, 1000.00) | 1000.00 (500.00, 1000.00) | 1000.00 (500.00, 1000.00) | Z=-1.65 | 0.100 | -0.269 | 1000.00 (500.00, 1000.00) | 1000.00 (500.00, 1000.00) | 1000.00 (500.00, 1000.00) | Z=-1.038 | 0.299 | 0.124 |
| Blood loss (ml), median (IQR) | 100.00 (50.00, 200.00) | 100.00 (50.00, 200.00) | 100.00 (50.00, 200.00) | Z=-0.71 | 0.478 | -0.061 | 100.00 (50.00, 200.00) | 100.00 (50.00, 200.00) | 100.00 (50.00, 200.00) | Z=-0.991 | 0.322 | -0.003 |
| Sufentanil dosage (ug), median (IQR) | 15.00 (0.00, 25.00) | 15.00 (0.00, 25.00) | 15.00 (10.00, 25.00) | Z=-1.09 | 0.275 | 0.063 | 15.00 (0.00, 25.00) | 15.00 (0.00, 25.00) | 15.00 (10.00, 25.00) | Z=-1.899 | 0.058 | 0.185 |
| Blood transfusion volume (ml), median (IQR) | 0.00 (0.00, 0.00) | 0.00 (0.00, 0.00) | 0.00 (0.00, 0.00) | Z=-0.49 | 0.621 | 0.036 | 0.00 (0.00, 0.00) | 0.00 (0.00, 0.00) | 0.00 (0.00, 0.00) | Z=-0.394 | 0.694 | -0.062 |
| PaCO_2_ (mmHg), median (IQR) | 40.00 (36.00, 45.00) | 39.00 (35.00, 44.00) | 42.00 (38.00, 46.00) | Z=-3.91 | <0.001 | 0.448 | 41.00 (37.00, 46.00) | 41.00 (37.00, 46.00) | 41.00 (38.00, 45.00) | Z=-0.054 | 0.957 | -0.021 |
| PaO_2_/FiO_2_ (mmHg), median (IQR) | 428.00 (259.50, 527.00) | 424.50 (260.00, 529.00) | 455.00 (257.00, 522.00) | Z=-0.77 | 0.440 | -0.028 | 450.00 (279.25, 526.00) | 412.00 (281.00, 528.00) | 456.00 (279.00, 522.00) | Z=-0.466 | 0.641 | -0.019 |
| HGB (g/L), median (IQR) | 123.00 (110.00, 135.00) | 123.00 (107.00, 136.00) | 123.00 (113.00, 134.00) | Z=-0.96 | 0.336 | 0.135 | 125.00 (113.00, 135.75) | 126.00 (113.00, 136.00) | 123.00 (113.00, 134.00) | Z=-0.672 | 0.502 | -0.050 |
| K^+^ (mmol/L), median (IQR) | 4.00 (3.60, 4.30) | 4.00 (3.70, 4.40) | 3.90 (3.60, 4.30) | Z=-3.62 | <0.001 | -0.356 | 3.90 (3.60, 4.20) | 3.90 (3.60, 4.20) | 3.90 (3.60, 4.30) | Z=-1.118 | 0.264 | -0.122 |
| **Postoperative data** | | | | | |  |  |  |  |  |  |  |
| Use of antagonistic drugs, n(%) | 137 (24.16) | 91 (24.07) | 46 (24.34) | χ²=0.00 | 0.945 | 0.006 | 76 (23.03) | 38 (23.03) | 38 (23.03) | χ²=0.000 | 1.000 | 0.000 |
| Postoperative pain score , median (IQR) | 3.00 (2.00, 3.00) | 3.00 (2.00, 3.00) | 3.00 (2.00, 3.00) | Z=-0.78 | 0.434 | 0.188 | 3.00 (2.00, 3.00) | 3.00 (2.00, 3.00) | 3.00 (2.00, 3.00) | Z=-0.912 | 0.362 | 0.058 |

PACU, post-anesthesia care unit; MAS, major abdominal surgery; SD, standard deviation; BMI, body mass index; DM, diabetes mellitus; CAD, coronary artery disease; ASA, American Society of Anesthesiologists; IQR, interquartile ranges; PaCO₂, partial pressure of carbon dioxide; PaO₂, partial pressure of oxygen; FiO₂, fraction of inspired oxygen; HGB, hemoglobin; PSM, propensity score matching; SMD, standardized mean difference

**Supplementary Table 2.** STROBE Statement—checklist of items for observational studies

| **#** | **Section**  **/Topic** | **Item (D;V)** | **Requirement** | **Location in Manuscript (to be filled)** |
| --- | --- | --- | --- | --- |
| **1** | Title / Abstract | Title 1 (D;V) | Identify the study as a prediction model development and validation study, including target population and outcome. | Title states “Development and Validation of Risk Factors Prediction Model for Hypoxemia”. |
| **2** | Title / Abstract | Abstract 2 (D;V) | Provide structured summary: objectives, design, setting, participants, sample size, predictors, outcome, statistical analysis, results, conclusion. | Abstract covers all; sample size and protective ORs updated. |
| **3a** | Introduction | Background 3a (D;V) | Explain medical background and rationale for developing/validating the prediction model. | Introduction . |
| **3b** | Introduction | Objectives 3b (D;V) | State specific objectives, including whether the study reports development, validation, or both. | Introduction. |
| **4a** | Methods | Source of Data 4a (D;V) | Describe study design and data sources, and clearly distinguish development and validation datasets. | Methods section (consecutive cohorts from two time periods). |
| **4b** | Methods | Key Dates 4b (D;V) | Specify key study dates: recruitment start/end, follow-up end. | Training: June 1, 2024 – Dec 31, 2024; Validation: Apr 1, 2025 – May 31, 2025. |
| **5a** | Methods | Setting 5a (D;V) | Describe study setting (e.g., single-center, multicenter), number and location of centers. | Single‑center (First Affiliated Hospital of Naval Medical University). |
| **5b** | Methods | Eligibility 5b (D;V) | Describe inclusion and exclusion criteria for participants. | Methods section (revised: added definition of severe respiratory disease). |
| **6a** | Methods | Outcome Definition 6a (D;V) | Clearly define the outcome, including measurement method and timing. | Hypoxemia = SpO₂ < 90% after extubation. |
| **6b** | Methods | Blinding Outcome 6b (D;V) | Report whether outcome assessment was blinded. | Retrospective design; Not applicable. |
| **7a** | Methods | Predictor Definition 7a (D;V) | Clearly define all predictors, including measurement methods and timing. | Added: PaO₂/FiO₂, HGB, K⁺ measured from first arterial blood gas upon PACU arrival, **before extubation**. |
| **7b** | Methods | Blinding Predictors 7b (D;V) | Report whether predictor assessment was blinded. | Retrospective; predictors extracted from medical records without knowledge of outcome. |
| **8** | Methods | Sample Size 8 (D;V) | Explain how sample size was determined. | when using the Wald method, an EPV greater than 10 is required to ensure the stability of the results. The final multivariable model included 4 predictors. Based on the previous experiment, the incidence of hypoxemia in the PACU after MAS was approximately 33.0%. With EPV set at 10, the required sample size was calculated as: (4 × 10) /0.33 ≈ 121 cases. After adding 10% to account for missing data, approximately 134 cases were needed. . |
| **9** | Methods | Missing Data 9 (D;V) | Describe handling of missing data (e.g., complete case, imputation). | for variables with a missing rate <5%, continuous variables with a normal distribution were imputed with the mean, non-normally distributed continuous variables with the median, and categorical variables with the mode; variables with missing rates between 5% and 20% were handled using multiple imputation; variables with missing rates ≥20% were excluded. |
| **10a** | Methods | Predictor Processing 10a (D;V) | Describe how continuous predictors were handled (cutoffs, transformations). | Added: optimal cutoffs determined by maximizing Youden index from ROC curves in training set. |
| **10b** | Methods | Model Building 10b (D;V) | Describe model building strategy (e.g., univariate screening, stepwise). | All variables with *P*<0.05 in univariate analysis entered simultaneously into multivariable model. Only those with P<0.05 in multivariable analysis retained. |
| **10c** | Methods | Model Performance 10c (D;V) | Describe measures used to evaluate model performance (discrimination, calibration). | AUC, calibration plot, H‑L test, DCA. |
| **10d** | Methods | Validation Method 10d (D) | Describe internal validation method (bootstrap, cross‑validation, temporal). | Temporal validation (different time period at same institution); 1000 bootstrap resampling for internal validation. |
| **11** | Methods | Risk Groups 11 (D;V) | Describe how risk groups were created. | Not applicable – nomogram provides individual risk probability; no arbitrary risk groups. |
| **12** | Results | Validation Comparison 12 (V) | For validation, report any differences between development and validation datasets. | Added Supplementary Table 1. comparing baseline characteristics; differences Limitations. |
| **13a** | Results | Participant Flow 13a (D;V) | Describe participant flow and number of outcome events. | Figure 1 flowchart; 112 events in training cohort. |
| **13b** | Results | Baseline Characteristics 13b (D;V) | Describe baseline characteristics and missing data. | Table 1 (training cohort) and Table 3 (comparison with validation cohort). |
| **13c** | Results | Validation Comparison 13c (V) | For validation, compare important variables between development and validation datasets. | Table 3. |
| **14a** | Results | Full Model 14a (D) | Present the full prediction model (regression coefficients, intercept). | Table 2 (ORs) and prediction formula. |
| **15** | Results | Model Performance 15 (D;V) | Report model performance metrics with confidence intervals. | AUC 0.88 (0.83-0.92) for training; AUC 0.89 (0.86-0.92) for validation. |
| **16** | Discussion | Limitations 16 (D;V) | Discuss study limitations. | Limitations section (expanded to include categorization bias, sample size, single‑center nature). |
| **17** | Discussion | Interpretation 17 (D;V) | Interpret results in light of limitations and objectives. | Discussion section. |
| **18** | Discussion | Implications 18 (D;V) | Discuss clinical implications and future research. | Discussion section. |
| **19** | Other | Protocol/Registration 19 (D;V) | Provide ethics approval and informed consent information. | Declarations section. |
| **20** | Other | Funding 20 (D;V) | Provide funding source. | Funding statement. |
| **21** | Other | Supplementary 21 (D;V) | Indicate where supplementary materials (e.g., TRIPOD checklist) are available. | Supplementary Table 2 (this checklist). |
| **22** | Other | Conflict of Interest 22 (D;V) | Provide conflict of interest statement. | Declarations section. |

Supplementary Table 3. Sensitivity analysis: multivariate logistic regression with all continuous predictors modeled as continuous variables

| Variables | β | S.E | Z | *P* | OR (95%CI) |
| --- | --- | --- | --- | --- | --- |
|  |  |  |  |  |  |
| Intercept | -1.89 | 2.40 | -0.79 | 0.432 | 0.15 (0.00-16.80) |
| Age | 0.05 | 0.02 | 3.23 | 0.001 | 1.05 (1.02-1.08) |
| BMI | 0.30 | 0.07 | 4.29 | <.001 | 1.35 (1.18-1.54) |
| Postoperative pain score | 0.12 | 0.07 | 1.67 | 0.096 | 1.13 (0.98-1.30) |
| PaO_2_/FiO_2_ | -0.01 | 0.00 | -6.15 | <.001 | 0.99 (0.99-0.99) |
| HGB | -0.08 | 0.01 | -6.05 | <.001 | 0.93 (0.90-0.95) |
| Smoking | 0.52 | 0.46 | 1.13 | 0.258 | 1.68 (0.68-4.12) |
| OR: Odds Ratio, CI: Confidence Interval | | | | | |
